# Transcriptomic Analysis of High and Low Lipid Droplet Deposition Subpopulations of Chicken Preadipocytes Based on SSC Sorting

**DOI:** 10.3390/ani16060885

**Published:** 2026-03-12

**Authors:** Boyu Wang, Yantao Li, Yake Wang, Jiayi Chen, Jiali Wang, Xiaoping Li, Zhenhui Li

**Affiliations:** 1 State Key Laboratory of Swine and Poultry Breeding Industry, Guangdong Laboratory of Lingnan Modern Agriculture, South China Agricultural University, Guangzhou 510642, China; bywang@stu.scau.edu.cn (B.W.); 20222024011@stu.scau.edu.cn (Y.L.); yk-2116@stu.scau.edu.cn (Y.W.); 13531122833@163.com (J.C.); 15713207180@163.com (J.W.); 13411168225@163.com (X.L.); 2 Guangdong Provincial Key Laboratory of Agro-Animal Genomics and Molecular Breeding, Key Laboratory of Chicken Genetics, Breeding and Reproduction, Ministry of Agriculture, College of Animal Science, South China Agricultural University, Guangzhou 510642, China

**Keywords:** chicken preadipocytes, flow cytometry, lipid metabolism, adipocyte metabolic heterogeneity

## Abstract

Fat deposition plays a crucial role in regulating the production performance and meat quality of broilers. Although the heterogeneity of mammalian adipocytes has been extensively studied, research on the molecular mechanisms underlying differences in lipid droplet accumulation in avian adipocytes remains limited. This study confirmed a significant positive correlation (R^2^ > 0.81, *p* < 0.001) between the SSC signal and lipid droplet content via fluorescence staining of lipid droplets, Oil Red O staining, and triglyceride (TG) quantification. Based on this, a label-free sorting strategy using SSC signals was established to sort differentiated chicken preadipocytes, obtaining high lipid droplet (H) and low lipid droplet (L) subpopulations, which were subsequently subjected to transcriptome sequencing and differential gene expression (DEG) analysis, followed by GO and KEGG enrichment analysis. The results indicated no significant differences in the expression of adipogenesis marker genes (*PPARG*, *LPL*, *CD36*, *PLIN1*, *PLIN2*) between the high lipid droplet (H) and low lipid droplet (L) groups, suggesting that both groups are at similar stages of differentiation. KEGG analysis revealed that both the H vs. NC and L vs. NC comparisons were enriched in common pathways, including the PPAR signaling pathway, ECM–receptor interaction, focal adhesion, cytokine–receptor interaction, and calcium–Apelin signaling pathway, suggesting that both groups of cells had activated the adipogenesis program. GO analysis showed that, in both H vs. NC and L vs. NC comparisons, differentially expressed genes (DEGs) were enriched in biological processes (BPs) related to cell adhesion, nucleosome assembly, chromatin remodeling, and receptor activity, as well as cellular components (CCs) such as the extracellular matrix, cytoskeleton, and nucleosome organization, indicating extensive gene reprogramming and activation of signaling transduction during differentiation. In the H vs. L comparison, enriched pathways included ABC transporters, ECM–receptor interaction, focal adhesion, gap junctions, microtubule-related processes, and neuroactive ligand–receptor interactions, involving lipid transmembrane transport, cytoskeleton stabilization, and signal transduction regulation, suggesting that high lipid droplet cells are more mature in lipid droplet transport, storage, and homeostasis maintenance. GO enrichment results further supported this conclusion, as H vs. L specifically enriched processes related to microtubule-related processes, cell cycle, and redox reactions (BPs), as well as chromosome organization, cytoskeleton, and motor activity (CC/MF), indicating that high lipid droplet cells maintain lipid droplet fusion and metabolic homeostasis via enhanced microtubule transport and antioxidant regulation. Differential gene analysis revealed that the L group upregulated genes associated with fatty acid synthesis and elongation (*ACACA*, *FASN*, *SCD*, *FADS2*, *ELOVL1*), cholesterol and isoprenoid biosynthesis (*HMGCR*, *SQLE*, *MSMO1*, *DHCR7*, *DHCR24*, *FDPS*, *LSS*), and fatty acid oxidation (*PPARA*, *PPARD*, *ACAD11*, *SIRT5*), reflecting a metabolic characteristic of concurrent lipid synthesis and mobilization; the H group, conversely, upregulated genes associated with lipid droplet formation and storage (*G0S2*, *MOGAT1*, *GPAT4*, *PLIN4*, *AUP1*), lipid transport (*ABCA1*, *ABCA2*, *ABCG1*, *OSBPL3*, *VLDLR*), and antioxidant defense (*GPX3*, *GPX4*, *HMOX1*), exhibiting a storage and homeostasis-oriented metabolic state. In the NC, L, and H groups, the expression of five genes—*GEM*, *SPP1*, *ABCA1*, *PDLIM3*, and *ITGA8*—showed a gradual increase, suggesting that these genes were associated with preadipocyte differentiation and lipid droplet deposition. In summary, although the high and low lipid droplet subpopulations of chicken preadipocytes exhibit similar differentiation states, they form distinct metabolic orientations. The L group is characterized by active lipid synthesis, fatty acid oxidation, and membrane lipid remodeling, while the H group predominantly features lipid droplet storage, lipid transport, and antioxidant homeostasis. This study highlights the molecular mechanisms underlying the metabolic heterogeneity of avian adipocytes and provides a theoretical basis for poultry fat deposition regulation and genetic improvement.

## Simple Summary

Fat deposition is a crucial economic trait that affects the production performance and meat quality of broilers. Moderate fat accumulation can enhance meat flavor, whereas excessive fat deposition decreases feed conversion rate and carcass yield. In this study, a label-free sorting method based on the Side Scatter (SSC) signal of flow cytometry was established to distinguish subpopulations of chicken preadipocytes with varying levels of lipid droplet deposition. The results demonstrated a significant positive correlation between the SSC signal and lipid droplet content (R^2^ > 0.81, *p* < 0.001), confirming that SSC is a reliable indicator for assessing lipid droplet accumulation in chicken preadipocytes. Further transcriptomic analysis revealed no significant difference in the expression of adipogenesis marker genes (*PPARG*, *LPL*, *CD36*, *PLIN1*, and *PLIN2*) between the high lipid droplet group (H group) and the low lipid droplet group (L group), suggesting that both are at similar differentiation levels. However, significant metabolic differences were observed between the two groups: the L group was primarily characterized by active lipid synthesis, fatty acid oxidation, and membrane lipid remodeling, whereas the H group was characterized by lipid droplet storage, lipid transport, and antioxidant homeostasis. This study suggests that these two types of cells are not at different differentiation stages but rather exhibit distinct metabolic orientations. This finding provides new insights into the molecular mechanisms underlying the metabolic heterogeneity of avian adipocytes.

## 1. Introduction

Fat deposition is a key economic trait influencing the production performance and meat quality of broilers. Moderate fat accumulation can enhance meat flavor and juiciness, while excessive fat deposition decreases feed conversion efficiency, lean meat yield, and consumer acceptability [1,2]. The formation of fat deposition primarily depends on the proliferation and differentiation of adipocytes, with preadipocyte differentiation and lipid droplet formation being crucial steps in adipose tissue development [3,4,5]. Therefore, understanding the molecular mechanisms regulating preadipocyte differentiation and lipid droplet accumulation holds significant theoretical and practical value for achieving precise control over fat deposition in broilers.

In mammals, numerous studies have shown that adipocytes within the same tissue exhibit considerable functional and metabolic heterogeneity. Researchers have identified several subpopulations of white adipocytes within the same fat depot in mice, differing in insulin sensitivity, lipid metabolism, and inflammatory responses [6]. The recent development of single-cell and single-nucleus transcriptomic technologies has further revealed various adipocyte subtypes with distinct transcriptional features in adipose tissue [7,8]. These subtypes not only differ in differentiation levels but also undergo dynamic remodeling due to developmental origin, environmental stimuli, or aging, particularly showing notable differences in de novo lipogenesis (DNL) activity [9]. These studies collectively demonstrate that adipocyte heterogeneity reflects not only varying differentiation stages but also functional states and metabolic orientations.

In contrast, research on adipocyte heterogeneity in poultry remains relatively limited. Most studies have focused on differences in tissue morphology, metabolic characteristics, and gene expression across different fat depots (abdominal fat, subcutaneous fat, and intramuscular fat) [10,11,12,13], or have explored adipogenesis-related genes and epigenetic regulation in mixed adipocyte systems [14,15,16,17,18]. However, within preadipocyte systems undergoing differentiation under the same induction conditions, the molecular mechanisms underlying differences in lipid droplet accumulation—whether stemming from varying degrees of differentiation or reflecting metabolic heterogeneity in adipocytes—remain underexplored.

In this study, a label-free sorting method was systematically validated and established based on the correlation between the side scatter (SSC) signal of flow cytometry and intracellular lipid droplet content in chicken preadipocytes. This method enables the differentiation of cell subpopulations with varying levels of lipid droplet deposition following differentiation. Transcriptome sequencing and functional enrichment analysis (GO and KEGG) were used to systematically compare the gene expression profiles of high lipid droplet (H) and low lipid droplet (L) subpopulations. This study aims to uncover the metabolic heterogeneity in chicken preadipocytes based on lipid droplet accumulation levels, providing new theoretical insights and technical support for the mechanistic understanding and genetic improvement of poultry fat deposition.

## 2. Materials and Methods

### 2.1. Cell Culture and Differentiation

In this study, the chicken preadipocyte cell line ICP2, kindly provided by Northeast Agricultural University, Harbin, China, was used [19], was used. The cells were cultured in complete DMEM/F12 medium containing 10% fetal bovine serum (FBS; Vivacell, Shanghai, China; C04002-500) and 1% penicillin–streptomycin (P/S; Gibco, Grand Island, NY, USA) at 37 °C in a 5% CO_2_ incubator. When the cells reached 60–80% confluence, differentiation was induced by culturing them in differentiation medium (DMEM/F12 + 10% FBS + 260 μM sodium oleate [Kunchuang, Wuxi, China; KC005]). The differentiation medium was replaced every 24 h until clear lipid droplet formation was observed.

### 2.2. Lipid Droplet Fluorescence Staining

In this study, lipid droplets in chicken preadipocytes were stained with a lipid droplet fluorescence dye, and fluorescence intensity was measured by flow cytometry to assess intracellular lipid droplet content. According to the instructions of the lipid droplet detection kit (Servicebio, Wuhan, China; G1905), the lipid droplet detection probe was mixed with the detection buffer at a 1:1000 ratio to prepare the working solution. Cells cultured in 12-well plates were collected, and the medium was discarded. The cells were gently washed twice with PBS. A total of 500 μL of the prepared working solution was added to each well, and the cells were incubated at 37 °C in the dark for 15 min. After incubation, the dye was discarded, and the cells were washed twice with PBS to remove unbound probe. Trypsin was added to each well to digest the cells at 37 °C until they detached. Digestion was terminated by adding complete medium, and the cells were gently pipetted to prepare a single-cell suspension. The cell suspension was analyzed using a flow cytometer, and fluorescence intensity was measured in the FITC channel. A minimum of 10,000 events were collected for each sample. Data analysis was performed using FlowJo v10.8.1 software, with fluorescence intensity used to evaluate lipid droplet content.

### 2.3. Oil Red O Staining and Quantification

To observe lipid accumulation and perform quantitative analysis, an Oil Red O staining kit (Solarbio, Beijing, China; G1262) was used to stain chicken preadipocytes. The kit includes a fixative and two working solutions, Oil Red A and Oil Red B. The procedure is as follows: After discarding the culture medium, the cells in 12-well plates were gently washed twice with PBS, and then fixed with the 4% fixative solution provided in the kit at room temperature for 30 min to preserve cell morphology. After fixation, the cells were washed twice with distilled water to remove any residual fixative. Next, according to the kit instructions, Oil Red A and Oil Red B were mixed at a 3:2 ratio, allowed to stand at room temperature for 10 min, and then filtered through a 0.45 μm membrane to prepare the Oil Red O working solution. A total of 300 μL of the working solution was added to each well, and the cells were incubated in the dark at room temperature for 15 min for staining. After staining, the cells were gently washed with 60% isopropanol for 20 s to remove unbound dye and then washed twice with distilled water. After washing, 500 μL of PBS was added to keep the cells moist for observation under an inverted microscope and to capture images of lipid droplet distribution. For quantitative analysis, after capturing images, the PBS was discarded, and 100% isopropanol was added to the wells and incubated at room temperature for 10 min to extract the bound Oil Red O dye. The absorbance of the extracted solution was measured at 510 nm using a spectrophotometer (OD_510_), reflecting the level of lipid accumulation within the cells.

### 2.4. Triglyceride (TG) Detection

To quantitatively analyze intracellular triglyceride (TG) content, the triglyceride detection kit (Solarbio, China; BC0620) was used according to the manufacturer’s instructions. The procedure was as follows: 5 × 10^6^ cells from each group were collected by centrifugation, and the supernatant was discarded. Then, 1 mL of Reagent 1 (a 1:1 (*v*/*v*) mixture of n-heptane and isopropanol) was added, and the mixture was thoroughly vortexed. Reagent 2 was then added (according to the kit instructions for blank and standard tubes), and the solution was vigorously shaken for 30 s. After standing for 3–5 min, the solution was shaken again for 30 s. This process was repeated three times. After standing at room temperature to allow phase separation, 30 μL of the upper solution was transferred to a new EP tube.

Following the kit instructions, Reagents 3 and 4 were added sequentially, mixed well, and the solution was incubated in a 65 °C water bath for 3 min. After cooling, Reagents 5 and 6 were added, mixed, and incubated in a 65 °C water bath for 15 min, followed by another cooling step. Finally, 200 μL of the reaction mixture was added to a 96-well plate, and absorbance (A) was measured at 420 nm using a spectrophotometer.

The TG content was calculated using the following formula: TG content = [(A_sample − A_blank)/(A_standard − A_blank)]/N. where A_sample, A_blank, and A_standard are the absorbance values of the sample, blank, and standard at 420 nm, respectively, and N is the cell number.

### 2.5. Flow Cytometry Analysis and Sorting

Flow cytometry analysis included Side Scatter (SSC) signal analysis of differentiated chicken preadipocytes and fluorescence intensity detection following lipid droplet fluorescence staining. These analyses were used to assess cell granularity and lipid droplet accumulation levels.

Before the experiment, the flow cytometer (BD Biosciences, San Jose, CA, USA) was turned on, and daily cleaning and laser calibration were performed according to standard procedures. Forward Scatter (FSC) and Side Scatter (SSC) parameters were set, and voltage and gain were adjusted to ensure clear distribution of the cell population on the scatter plot. Specifically, the FSC voltage was set within the range of 300–400 V (optimized according to the cell size aggregation signal), and the SSC voltage was set within the range of 250–400 V (adjusted according to the granularity distribution). Unstained and lipid droplet fluorescence-stained cell suspensions were analyzed, and fluorescence signals were collected through the FITC channel (Ex/Em ≈ 488/530 nm). A minimum of 10,000 cell events were collected for each sample to ensure the reliability of the statistical results. Flow cytometry data were analyzed using FlowJo v10.8.1 software.

The target cell population was selected using FSC vs. SSC scatter plots, and debris and dead cells were excluded. Specifically, a gate was set around the main peak population to exclude debris in the lower-left corner (low FSC/low SSC) and aggregates in the upper-right corner (high SSC/high FSC). The average SSC value and average fluorescence intensity of each group were calculated and recorded.

For the cell sorting experiment, differentiated chicken preadipocytes on day 4 were selected, and sorting gates were set based on SSC values: the low SSC group consisted of the lowest 0–10% of the cell population, and the high SSC group consisted of the highest 90–100% of the cell population. Sorting was performed in low-speed mode (sheath pressure < 20 psi) to ensure cell viability and sorting purity. The sorted cells were immediately used for subsequent experiments.

### 2.6. Transcriptome Sequencing

To analyze the transcriptomic characteristics of high lipid droplet (H), low lipid droplet (L), and undifferentiated (NC) cell groups (n = 3 per group), total RNA was extracted, followed by quality assessment and library construction for sequencing. After RNA extraction, RNA integrity and purity were assessed using the Agilent 2100 Bioanalyzer (Agilent Technologies, Santa Clara, CA, USA). Qualified samples were processed using oligo(dT) magnetic beads to enrich mRNA, and cDNA was synthesized using M-MLV Reverse Transcriptase (Promega, Madison, WI, USA). Subsequently, the cDNA library was purified, the ends were repaired, an A-tail was added, and adapter ligation was performed. AMPure XP magnetic beads (Beckman Coulter, Brea, CA, USA) were used to select fragments of appropriate size, and PCR amplification was carried out to complete library construction. Library quality and insert fragment length were verified using a Qubit 2.0 Fluorometer (Thermo Fisher Scientific, Waltham, MA, USA) and the Agilent 2100 Bioanalyzer, respectively. Sequencing was performed on the Illumina NovaSeq 6000 platform (Illumina, San Diego, CA, USA) using paired-end 150 bp sequencing. During the data quality control stage, fastp (version 0.19.7) software was used to remove adapter sequences, low-quality reads, and sequences containing unknown bases (N) to ensure the reliability of subsequent analysis data.

High-quality data were aligned to the chicken reference genome (Ensembl GRCg6a, Release 106) using HISAT2 (v2.2.1). Gene expression levels (FPKM/TPM) were obtained using featureCounts (v2.0.1), and differential expression analysis was performed with DESeq2 (v1.34.0). The criteria for screening differential genes were |log_2_FC| ≥ 1 and *p* < 0.05. Differentially expressed genes (DEGs) were subjected to Gene Ontology (GO) and Kyoto Encyclopedia of Genes and Genomes (KEGG) functional enrichment analysis, with a significance threshold set at *p* < 0.05.

### 2.7. RNA Extraction and RT-qPCR

Total RNA was extracted from cells using TRIzol reagent (Invitrogen, Grand Island, NY, USA) in accordance with the manufacturer’s instructions. Complementary DNA (cDNA) was synthesized using reverse transcriptase (Novizan, Nanjing, China), with β-actin used as the internal reference gene for normalization of gene expression levels. The synthesized cDNA served as the template for PCR amplification under the following conditions: pre-denaturation at 95 °C for 5 min; 40 cycles of denaturation at 95 °C for 30 s, annealing at 58 °C for 30 s, and extension at 72 °C for 1 min; followed by a final extension at 72 °C for 10 min. RT-qPCR was performed using an Applied Biosystems QuantStudio 5 system (Waltham, MA, USA), and relative gene expression levels were calculated using the 2^−ΔΔCt^ method. The primer sequences used for qPCR are listed in Table 1 and were designed based on gene sequences obtained from the NCBI database using Primer 5.0 software.

### 2.8. Data Statistical Analysis

Data analysis in this study was performed using GraphPad Prism software (Version 5.0, Harvey Motulsky, GraphPad Software, San Diego, CA, USA). The results are presented as mean ± standard error (Mean ± SE). Differences between two groups were analyzed using a two-tailed Student’s *t*-test. A *p*-value of <0.05 was considered statistically significant. Each experimental group included three biological replicates, and all experiments were independently repeated at least three times to ensure data reliability.

## 3. Results

### 3.1. Correlation Verification of Side Scatter (SSC) as an Indicator of Lipid Droplet Deposition

**Figure 1:**
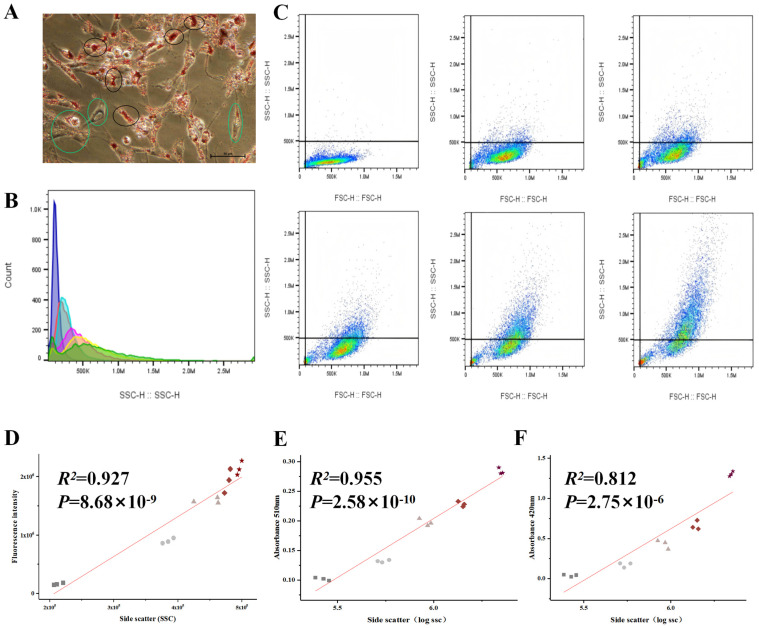
Correlation Analysis Between Differentiation Phenotype and SSC of Chicken Preadipocytes. (**A**): Oil Red O staining of chicken preadipocytes on day 4 of differentiation. The cells in the green box indicate cells with fewer lipid droplets, whereas the cells in the black box indicate cells with more lipid droplets. (**B**): Bar graph of SSC values of chicken preadipocytes from days 0 to 5 of differentiation. The colors purple, red, blue, pink, yellow, and green represent SSC values on differentiation days 0, 1, 2, 3, 4, and 5, respectively. (**C**): FSC vs SSC scatter plot of chicken preadipocytes from days 0 to 5 of differentiation. In the scatter plot, blue indicates fewer cells, while green indicates a higher number of cells. (**D**): Correlation analysis between SSC values and lipid droplet-specific fluorescence intensity. Squares, circles, triangles, diamonds, and pentagrams represent chicken preadipocytes at differentiation days 0, 1, 2, 3, and 4, respectively. (**E**): Correlation analysis between SSC values and the absorbance (OD) at 510 nm of the Oil Red O extract. Squares, circles, triangles, diamonds, and pentagrams represent chicken preadipocytes differentiated for 4 days and treated with sodium oleate at concentrations of 0, 100, 200, 400, and 800 μM, respectively. (**F**): Correlation analysis between SSC values and triglyceride (TG) content. Symbols are the same as in (**E**).

During the differentiation of chicken preadipocytes, lipid droplet deposition exhibited significant heterogeneity between cells. On day 4 of differentiation, even within the same culture dish, noticeable differences in the size and number of lipid droplets were observed (black circles represent high lipid droplet cells, green circles represent low lipid droplet cells; Figure 1A), suggesting that lipid droplet accumulation varies within the cell population. The SSC signal from days 0 to 5 of differentiation was measured, and the results showed that the SSC value gradually increased as differentiation progressed (Figure 1B,C), indicating that the complexity of intracellular structures increased with lipid droplet formation.

To verify whether the changes in the SSC signal were directly correlated with lipid droplet deposition, both SSC values and lipid droplet-specific fluorescence signals were measured in samples from days 0 to 4 of differentiation, followed by correlation analysis. The results showed a strong positive correlation between SSC values and lipid droplet fluorescence intensity (R^2^ = 0.927, *p* < 0.001; Figure 1D), indicating that SSC values can effectively reflect lipid droplet accumulation levels.

Furthermore, to strengthen the validation, preadipocytes were treated with different concentrations of sodium oleate (0, 100, 200, 400, 800 μM) for 4 days, and the SSC mean values, OD values of the Oil Red O staining extracts, and triglyceride (TG) content were measured and correlated. The results showed a very strong positive correlation between SSC values and the absorbance at 510 nm of the Oil Red O extract (R^2^ = 0.955, *p* < 0.001; Figure 1E), and a significant positive correlation with TG content (R^2^ = 0.812, *p* < 0.001; Figure 1F).

In summary, the SSC signal can serve as a reliable indicator of lipid droplet accumulation in chicken preadipocytes, providing experimental support for subsequent sorting based on flow cytometry features.

### 3.2. Phenotypic and Transcriptomic Differences in SSC-Defined Chicken Preadipocyte Subpopulations

**Figure 2:**
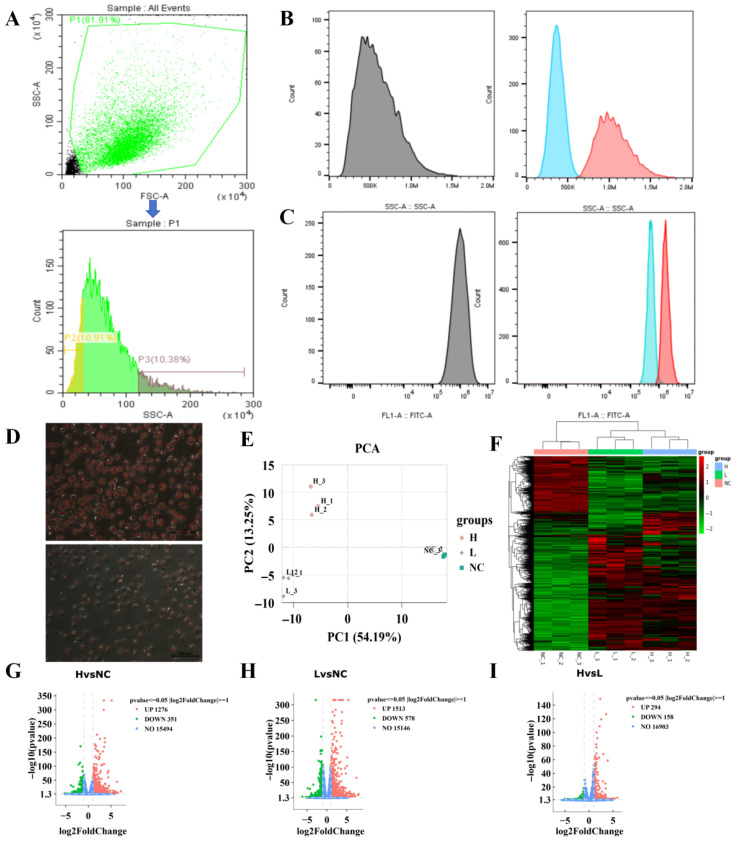
Phenotypic Validation and Transcriptomic Analysis of Chicken Preadipocytes Based on SSC Sorting. The experimental samples consisted of chicken preadipocytes induced to differentiate with sodium oleate for 4 days. Cells were sorted by flow cytometry based on the SSC parameter to obtain high-SSC and low-SSC subpopulations. After sorting, the cells were characterized by SSC values, lipid droplet–specific fluorescence signals, and Oil Red O staining. (**A**): Schematic illustration of SSC-based cell sorting. P1 represents the cell population with optimal cell status (cells within the green box in the upper panel), P2 represents the low-SSC population (cells within the yellow box), and P3 represents the high-SSC population (cells within the dark green box). (**B**): SSC signal distribution of the sorted high-SSC and low-SSC cell populations. Gray, blue, and red histograms represent the SSC distributions of cells before sorting, sorted low-SSC cells, and sorted high-SSC cells, respectively. After sorting, a clear separation of SSC signals between the high- and low-SSC subpopulations was observed. (**C**): Lipid droplet–specific fluorescence signal distribution of the sorted high-SSC and low-SSC cell populations. Gray, blue, and red histograms represent the fluorescence signal distributions of cells before sorting, sorted low-SSC cells, and sorted high-SSC cells, respectively. After sorting, a clear separation of lipid droplet fluorescence signals between the high- and low-SSC subpopulations was observed. (**D**): Oil Red O staining of the sorted high-SSC and low-SSC cell populations, indicating intracellular lipid accumulation. (**E**): Principal Component Analysis (PCA) of transcriptomic profiles from the three groups (H, L, and NC), with three biological replicates per group (n = 3). (**F**): Hierarchical clustering heatmap of differentially expressed genes (DEGs) among the three groups, with color intensity indicating relative gene expression levels. (**G**): Volcano plot of DEGs between the H group and the NC group. (**H**): Volcano plot of DEGs between the L group and the NC group. (**I**): Volcano plot of DEGs between the H group and the L group. The threshold for differential genes is |log2FC| ≥ 1 and *p* < 0.05. The vertical gray dashed lines indicate |log2FC| = 1, and the horizontal gray dashed line indicates *p* = 0.05.

Based on the Side Scatter (SSC) parameters of chicken preadipocytes on day 4 of differentiation, flow cytometry sorting was performed (Figure 2A), yielding cells with high SSC values (H group) and low SSC values (L group), with undifferentiated cells serving as the control group (NC group). Post-sorting phenotypic validation showed significant separation between the H and L groups in both SSC values and lipid droplet-specific fluorescence intensity (Figure 2B,C), and the results of Oil Red O staining were consistent with these findings (Figure 2D), further supporting the validity of the sorting groups.

Subsequently, transcriptome sequencing (n = 3) was performed on the three groups to investigate the molecular regulatory mechanisms involved in adipogenesis and lipid droplet deposition. Principal Component Analysis (PCA) revealed significant transcriptomic differences among the three groups (Figure 2E), and the clustering heatmap also revealed obvious differences in gene expression profiles among the three groups (Figure 2F). Based on the criteria of |log_2_FC| ≥ 1 and *p* < 0.05, Volcano plot analysis identified a total of 1627 differentially expressed genes (DEGs) in H vs. NC, 2091 DEGs in L vs. NC, and 452 DEGs in H vs. L (Figure 2G–I). These results indicate that gene expression within the cells is subject to complex and fine-tuned regulation during adipogenesis and across different levels of lipid droplet accumulation.

### 3.3. GO and KEGG Enrichment Features of DEGs Associated with High and Low Lipid Droplet Deposition

To further investigate the molecular mechanisms underlying the differences in lipid droplet deposition, KEGG and GO enrichment analyses were performed. The analyses were conducted on the differentially expressed genes (DEGs) identified from the H vs. NC, L vs. NC, and H vs. L comparisons.

**Figure 3:**
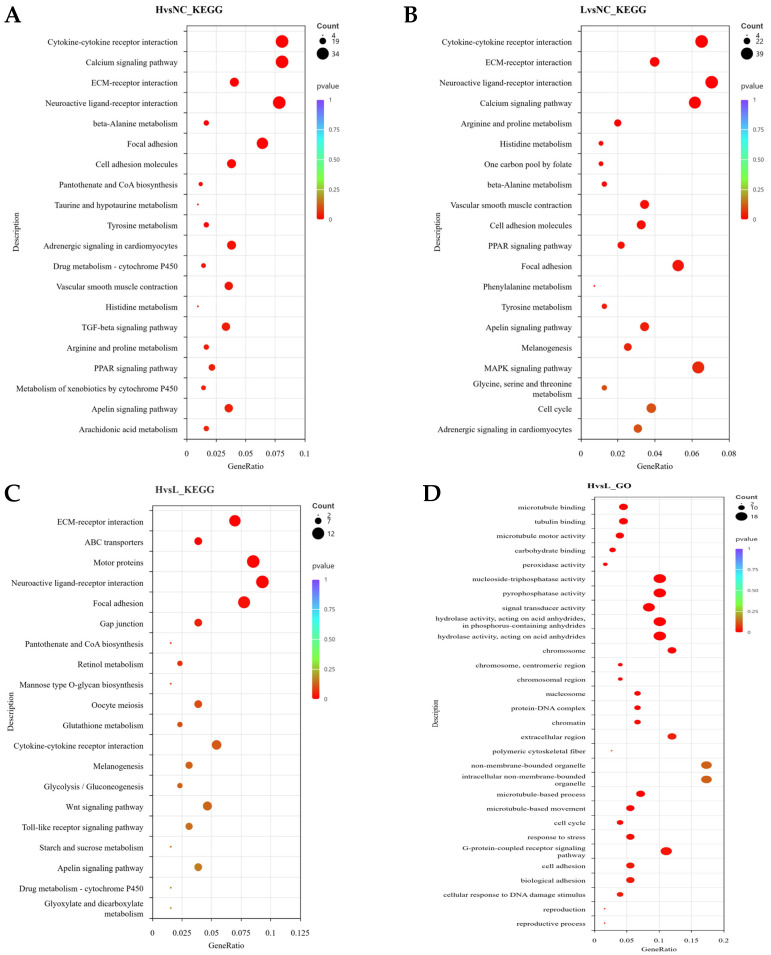
KEGG and GO Enrichment Analysis of Differential Genes Among the Three Groups. (**A**): KEGG analysis of H vs. NC. (**B**): KEGG analysis of L vs. NC. (**C**): KEGG analysis of H vs. L. (**D**): GO analysis results of H vs. L.

In the KEGG pathway analysis, both the H vs. NC and L vs. NC groups were significantly enriched in several signaling pathways related to adipocyte differentiation and metabolism, including the PPAR signaling pathway, ECM–receptor interaction, focal adhesion, cytokine–receptor interaction, and calcium–Apelin signaling pathway (Figure 3A,B). These commonly enriched pathways suggest that both differentiated cell groups have activated the adipocyte differentiation program. In the H vs. L comparison, significantly enriched pathways included ABC transporter pathways, ECM–receptor interaction, focal adhesion, gap junctions, microtubule-related processes, and neuroactive ligand–receptor interactions (Figure 3C). These pathways are primarily involved in lipid transmembrane transport, cell structural stability, and signal transduction regulation, indicating that H group cells exhibit higher maturity and activity in maintaining lipid droplet homeostasis, lipid transport, and signal response.

The results of GO enrichment analysis corroborated the KEGG findings. In the H vs. L comparison, the enriched biological processes (BP) mainly included microtubule-related processes, cell cycle, and stress responses; cellular components (CC) were enriched in chromosomes, nucleosomes, and cytoskeleton structures; and molecular functions (MF) were enriched in microtubule binding, motor protein activity, and peroxidase activity (Figure 3D).These results suggest that high lipid droplet cells (H group) may achieve efficient lipid droplet transport, fusion, and antioxidant homeostasis by enhancing the cytoskeleton–microtubule transport system and redox regulation mechanisms, thereby supporting higher levels of lipid droplet deposition and metabolic activity.

### 3.4. Molecular Features of Preadipocytes with High and Low Lipid Droplet Accumulation

**Figure 4:**
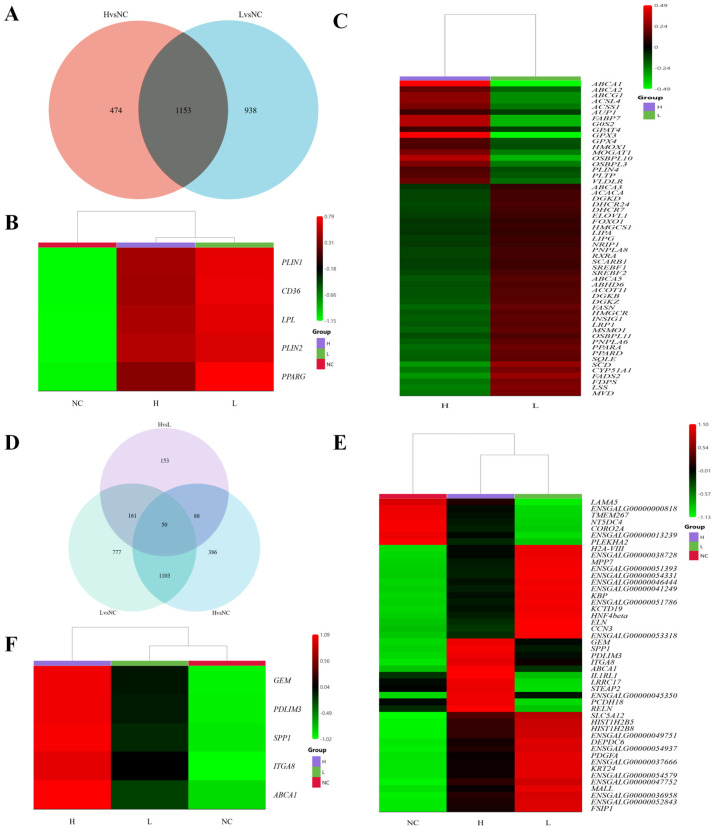
Transcriptome Differential Gene Analysis. (**A**): Venn diagram of differentially expressed genes (DEGs) in H vs. NC and L vs. NC comparisons. (**B**): Transcriptomic expression levels of preadipocyte differentiation marker genes across the three groups (NC, L, H). (**C**): The 55 lipid metabolism-related DEGs in the H vs L comparison. (**D**): Venn diagram of DEGs across the three groups. (**E**): List of common DEGs across the three groups. (**F**): Genes with continuously increasing mRNA expression in the NC, L, and H groups.

To explore the molecular sources of differences in lipid droplet deposition, Venn diagram analysis was performed on the differentially expressed genes (DEGs) from the H vs. NC and L vs. NC comparisons. The results revealed 1153 common DEGs between the two groups (Figure 4A). Among these, core marker genes for preadipocyte differentiation (*PPARG*, *LPL*, *PLIN1*, *PLIN2*, *CD36*) showed significant differences in both the H vs. NC and L vs. NC comparisons, but no significant changes were observed in the H vs. L comparison (Figure 4B), suggesting that the differentiation levels of the two groups of cells are similar.

To further explore molecular differences between high and low lipid droplet deposition cells, a relatively less stringent DEG threshold (|log_2_FC| ≥ 0 and *p* < 0.05) was selectively applied to the H vs. L comparison to capture genes with modest but potentially biologically relevant expression changes. Based on this criterion, a total of 55 lipid metabolism–related genes were identified (Figure 4C). Functional category analysis revealed that, in the low lipid droplet group (L), genes related to de novo fatty acid synthesis and unsaturation/chain elongation (*ACACA*, *FASN*, *SCD*, *FADS2*, *ELOVL1*) were significantly upregulated. Additionally, genes involved in cholesterol and isoprenoid biosynthesis pathways (*HMGCR*, *HMGCS1*, *SQLE*, *CYP51A1*, *MSMO1*, *DHCR7*, *DHCR24*, *FDPS*, *MVD*, *LSS*) were also significantly enhanced. Several transcriptional regulators and nuclear receptors (*SREBF1*, *SREBF2*, *INSIG1*, *PPARA*, *PPARD*, *RXRA*, *FOXO1*, *NRIP1*) were upregulated, reflecting the widespread activation of lipid synthesis networks and fatty acid oxidation. Concurrently, genes involved in membrane lipid remodeling (*DGKZ*, *DGKB*, *DGKD*, *PNPLA6*, *PNPLA8*, *ABHD6*, *ACOT11*) and lipid uptake (*LRP1*, *SCARB1*, *LIPA*, *LIPG*, *ABCA3*, *ABCA5*, *OSBPL11*) also showed an upregulation trend. These results indicate that low lipid droplet cells are in a state of active lipid synthesis, membrane lipid dynamic remodeling, and increased fatty acid oxidation.

In contrast, the high lipid droplet group (H) was characterized by a significant upregulation of genes associated with lipid droplet storage and triglyceride synthesis (*G0S2*, *MOGAT1*, *GPAT4*, *PLIN4*, *AUP1*). Additionally, genes involved in lipid transport and cholesterol efflux (*VLDLR*, *PLTP*, *ABCA1*, *ABCG1*, *ABCA2*, *OSBPL3*, *OSBPL10*, *FABP7*) were upregulated, suggesting that lipid droplet storage and efflux processes tend toward equilibrium. Moreover, the upregulation of genes involved in fatty acid activation and acetate utilization (*ACSL4*, *ACSS1*) and antioxidant and stress response genes (*GPX3*, *GPX4*, *HMOX1*) indicated that high lipid droplet cells maintain high lipid loads through enhanced redox homeostasis and lipid droplet protection mechanisms.

To further identify key genes involved in chicken preadipocyte differentiation and lipid droplet deposition, a Venn analysis of the DEGs across the three groups was performed. The results revealed 50 common DEGs across all three groups (Figure 4D,E). Interestingly, the mRNA expression of five genes—*GEM*, *PDLIM3*, *SPP1*, *ITGA8*, and *ABCA1*—showed a continuous increase in the NC, L, and H groups (Figure 4F), suggesting that these five genes may play an important role in the differentiation and lipid droplet deposition of chicken preadipocytes.

### 3.5. Verification of Transcriptomic Data Using RT-qPCR

**Figure 5:**
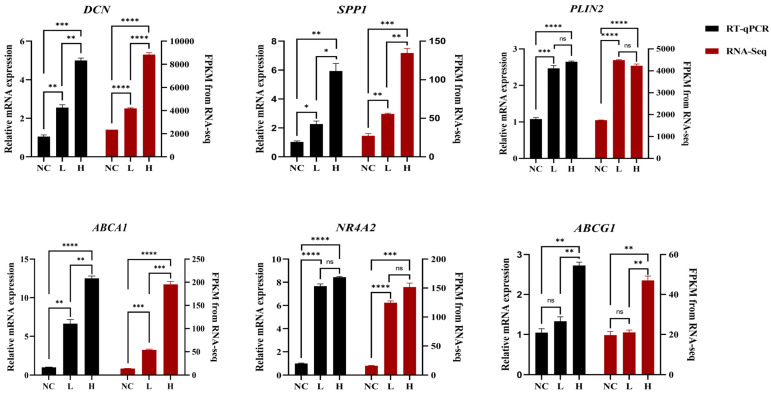
RNA-seq data were validated by RT-qPCR. The black bars on the left *y*-axis represent the relative expression levels determined by RT-qPCR, while the red bars on the right *y*-axis represent the FPKM values obtained from RNA-seq. All data are presented as the mean of three biological replicates, with error bars indicating the standard error (SE), and all values were normalized. Statistical significance is indicated as follows: ns, not significant; * *p* < 0.05; ** *p* < 0.01; *** *p* < 0.001; **** *p* < 0.0001.

To verify the accuracy of the RNA-seq data, total RNA was extracted from day 4 differentiated chicken preadipocytes sorted by flow cytometry into high- and low-SSC populations (H and L groups), as well as from the undifferentiated control group (NC). The expression levels of six representative differentially expressed genes identified from the transcriptomic analysis (*DCN*, *SPP1*, *PLIN2*, *ABCA1*, *NR4A2*, and *ABCG1*) were then validated by RT-qPCR. The gene expression patterns obtained from RT-qPCR analysis were highly consistent with the RNA-seq results (Figure 5), thereby confirming the reliability of the RNA-seq data. This concordance further supports the validity and robustness of the transcriptomic findings.

## 4. Discussion

Chicken meat is one of the most widely consumed animal protein sources worldwide and constitutes an important component of the human diet [20]. However, fat deposition, particularly abdominal fat deposition (AFD), has profound effects on meat quality and production performance in broiler chickens. Excessive fat accumulation not only reduces feed conversion efficiency but also markedly decreases carcass lean meat yield [21,22], and may further impair reproductive performance in breeder chickens by reducing egg production, fertilization rate, and hatchability [23]. Therefore, effective regulation of fat deposition, especially the excessive accumulation of abdominal fat, has become a critical challenge in global broiler breeding and production.

In the present study, side scatter (SSC) signals obtained from flow cytometry were, for the first time, integrated with transcriptomic analysis to systematically characterize transcriptional differences in chicken preadipocytes exhibiting distinct levels of lipid droplet accumulation, defined as high lipid droplet (H) and low lipid droplet (L) groups. The results demonstrated a strong positive correlation between SSC intensity and intracellular lipid droplet content in chicken preadipocytes (R^2^ > 0.81, *p* < 0.001), enabling accurate separation of H and L cell populations based solely on SSC signals. These findings provide direct evidence supporting the reliability of SSC as an indicator of lipid droplet burden. Previous studies in mammalian adipocytes have shown that flow cytometry can effectively quantify intracellular neutral lipid content, and that SSC signals, particularly when combined with lipid droplet–specific fluorescent dyes, allow quantitative assessment of lipid droplet levels [24,25]. Our study further confirms the accuracy and applicability of SSC-based evaluation of lipid droplet content and extends its utility to avian adipocyte research.

Intercellular heterogeneity in lipid droplet accumulation has been extensively documented in mammalian systems. For example, primary mouse hepatocytes have been shown to consist of subpopulations with distinct levels of lipid droplet accumulation, which exhibit marked differences in metabolic activity and cellular function. Such heterogeneity is thought to alleviate lipotoxicity by redistributing lipid load and thereby protecting cells from excessive lipid-induced damage [26]. Similarly, heterogeneity in lipid droplet accumulation has been observed during adipogenesis in mice, reflecting the dynamic coexistence of adipocyte subpopulations with different physiological states [27]. Comparable phenomena have also been reported in lipid droplet induction experiments involving clonal yeast, fibroblasts, and tumor cell lines [28,29,30]. Consistent with these observations, our study revealed pronounced heterogeneity in lipid droplet accumulation among chicken preadipocytes under sodium oleate–induced differentiation conditions. These findings suggest that intercellular heterogeneity in lipid droplet deposition is likely a conserved biological feature, although its manifestation and extent may vary across species and cell types.

Previous studies have identified three functionally distinct adipocyte subtypes within white adipose tissue: lipogenic adipocytes (LGA), lipid-scavenging adipocytes (LSA), and stressed lipid-scavenging adipocytes (SLSA). Lipids in the LGA subtype are primarily derived from de novo lipogenesis, whereas those in LSA and SLSA are more likely acquired through exogenous lipid uptake. Moreover, high-fat and high-sugar diet–induced obesity has been shown to drive a transition of adipocytes from the LGA phenotype toward LSA and SLSA states, accompanied by widespread downregulation of lipogenesis-related genes [31]. In agreement with this concept, transcriptomic analysis in the present study revealed significant upregulation of genes involved in fatty acid synthesis and elongation (*ACACA*, *FASN*, *SCD*, *FADS2*, *ELOVL1*) in the L group, whereas genes associated with lipid transport and uptake (*ABCA1*, *ABCA2*, *ABCG1*, *OSBPL3*, *VLDLR*) were predominantly upregulated in the H group. These results suggest that lipid accumulation in low lipid droplet cells may rely primarily on de novo lipogenesis, while high lipid droplet cells preferentially achieve rapid lipid droplet expansion through enhanced lipid uptake and transport.

In addition, previous research has indicated that, in mouse hepatocytes, cells with high lipid droplet accumulation exhibit stronger lipid uptake capacity and elevated levels of reactive oxygen species (ROS) compared with cells containing fewer lipid droplets. Notably, high lipid droplet–laden cells may reduce ROS levels in low lipid droplet cells through as-yet-unknown mechanisms, thereby exerting a protective effect [26]. In the present study, the significant upregulation of antioxidant defense-related genes (*GPX3*, *GPX4*, *HMOX1*) in the H group suggests that cells with high lipid droplet accumulation may depend on enhanced antioxidant responses to mitigate oxidative stress generated during lipid storage.

Microtubules, as essential components of the cytoskeleton, have been shown to participate in lipid droplet dynamics through direct interactions with lipid droplets, influencing their aggregation, distribution, and stability, and thus playing a critical role in lipid storage [32]. Recent studies further demonstrate that microtubules not only serve as major intracellular transport tracks but also regulate lipid droplet movement, fusion, and homeostasis, thereby contributing to lipid droplet storage and accumulation [33]. These findings are highly consistent with the significant enrichment of microtubule-related pathways observed in high lipid droplet cells in this study, indicating that microtubule-associated processes may be crucial for maintaining lipid droplet homeostasis in cells with high lipid content.

In summary, intercellular heterogeneity in lipid droplet accumulation is a widespread phenomenon across diverse cell types. It has been proposed that, within a cell population, a small subset of cells in a high lipid droplet state may bear a disproportionate lipid load and oxidative stress, thereby buffering other cells from lipotoxicity and oxidative damage at the population level [26]. Our findings demonstrate that chicken preadipocytes likewise exhibit pronounced heterogeneity in lipid droplet accumulation, which may be closely associated with transcriptional regulation of lipid synthesis and transport pathways, antioxidant defense mechanisms, and cytoskeleton-related processes.

Based on these observations, it can be hypothesized that the formation of abdominal fat deposition in broiler chickens involves the coordinated action of multiple adipocyte subpopulations with distinct functional states. However, research on adipocyte heterogeneity in chickens is currently limited, and most of our inferences are still based on studies conducted in mammalian models, which may present certain limitations when applied to avian species. Although studies on adipocyte heterogeneity in mammals have provided valuable insights, it remains unclear whether these mechanisms can be directly applied to poultry species, such as chickens. Future research focusing on nutritional or molecular regulatory strategies, elucidating the key factors influencing lipid droplet heterogeneity, and investigating the dynamic proportions of adipocyte subpopulations with varying lipid droplet content could provide new theoretical perspectives and research directions for understanding and precisely controlling abdominal fat deposition in broiler chickens.

## 5. Conclusions

This study reveals the metabolic heterogeneity of chicken preadipocytes at different lipid droplet deposition levels through flow cytometry Side Scatter (SSC) signals and transcriptomic analysis. Although high lipid droplet (H group) and low lipid droplet (L group) cells show similar expression of adipogenesis marker genes, they exhibit significant differences in metabolic functions and regulatory pathways. L group cells are more inclined toward lipid synthesis and fatty acid oxidation, whereas H group cells are primarily involved in lipid droplet storage and the maintenance of antioxidant homeostasis. These results suggest that differences in lipid droplet deposition stem from heterogeneity in cellular function and metabolic orientation rather than differences in differentiation stages. This study provides new insights into avian adipocyte metabolic heterogeneity and offers a fresh perspective for future research on fat deposition regulation and genetic improvement.

## Figures and Tables

**Table 1:** Primers used to validate RNA-seq accuracy.

Primer Name	Primer Sequences
*DCN*-F	5′-AGTTGAGGAAGGCAGT-3′
*DCN*-R	5′-AGGGATGGAGGAAGAC-3′
*SPP1*-F	5′-GGCATTTCTTTGCTTGTG-3′
*SPP1*-R	5′-CCTGGGGTCGTATTTTTC-3′
*PLIN2*-F	5′-TCTTGGGAAGTCGTGTGGTG-3′
*PLIN2*-R	5′-CACGTGCACGGAACTTTGAA-3′
*ABCA1*-F	5′-TACAATCCAAAATCGCTCAGT-3′
*ABCA1*-R	5′-GAACATCACCTCTTGCCTCAT-3′
*NR4A2*-F	5′-CCATCGTTGAGTTTTC-3′
*NR4A2*-R	5′-GCTTGGGTTCTTTGAG-3′
*ABCG1*-F	5′-TCTCCTACCTACCACGC-3′
*ABCG1*-R	5′-CTGCTGAACTTCCCTGA-3′
*β-actin*-F	5′-CCAGCCATCTTTCTTGGGTA-3′
*β-actin*-R	5′-ATGCCAGGGTACATTGTGGT-3′

## Data Availability

The data presented in this study are available on request from the corresponding author. The data are not publicly available due to the large size of the datasets.
